# MicroRNAs miR-142-5p, miR-150-5p, miR-320a-3p, and miR-4433b-5p in Serum and Tissue: Potential Biomarkers in Sporadic Breast Cancer

**DOI:** 10.3389/fgene.2022.865472

**Published:** 2022-06-30

**Authors:** Tamyres Mingorance Carvalho, Guillermo Ortiz Brasil, Tayana Schultz Jucoski, Douglas Adamoski, Rubens Silveira de Lima, Cleverton C. Spautz, Karina Furlan Anselmi, Patricia Midori Murobushi Ozawa, Iglenir João Cavalli, Jaqueline Carvalho de Oliveira, Daniela Fiori Gradia, Enilze Maria de Souza Fonseca Ribeiro

**Affiliations:** ^1^ Laboratory of Human Cytogenetics and Oncogenetics, Postgraduate Program in Genetics, Department of Genetics, Federal University of Paraná (UFPR), Curitiba, Brazil; ^2^ Brazilian Biosciences National Laboratory (LNBio), Brazilian Center for Research in Energy and Materials (CNPEM), Sao Paulo, Brazil; ^3^ Breast Disease Center, Hospital Nossa Senhora das Graças, Curitiba, Brazil; ^4^ Department of Cell and Developmental Biology, Vanderbilt University School of Medicine, Nashville, TN, United States

**Keywords:** breast cancer, miR-142-5p, miR-150-5p, miR-320a, miR-4433b-5p, biomarkers

## Abstract

Breast cancer (BC) is a heterogeneous disease, and establishing biomarkers is essential to patient management. We previously described that extracellular vesicle–derived miRNAs (EV-miRNAs) miR-142-5p, miR-150-5p, miR-320a, and miR-4433b-5p in serum discriminated BC from control samples, either alone or combined in a panel. Using these previously described markers, we intend to evaluate whether the same markers identified in EVs are also potential biomarkers in tissue and serum. Expression analysis using RT-qPCR was performed using serum of 67 breast cancer patients (BC-S), 19 serum controls (CT), 83 fresh tumor tissues (BC-T), and 29 adjacent nontumor tissue samples (NT). In addition, analysis from The Cancer Genome Atlas (TCGA) data (832 BC-T and 136 NT) was performed. In all comparisons, we found concordant high expression levels of miR-320a and miR-4433b-5p in BC-S compared to CT in both EVs and cell-free miRNAs (cf-miRNAs). Although miR-150-5p and miR-142-5p were not found to be differentially expressed in serum, panels including these miRNAs improved sensitivity and specificity, supporting our previous findings in EVs. Fresh tissue and data from the TCGA database had, in most comparisons, an opposite behavior when compared to serum and EVs: lower levels of all miRNAs in BC-T than those in NT samples. TCGA analyses revealed reduced expression levels of miR-150-5p and miR-320a-3p in BC-T than those in NT samples and the overexpression of miR-142-5p in BC-T, unlike our RT-qPCR results from tissue in the Brazilian cohort. The fresh tissue analysis showed that all miRNAs individually could discriminate between BC-T and NT in the Brazilian cohort, with high sensitivity and sensibility. Furthermore, combining panels showed higher AUC values and improved sensitivity and specificity. In addition, lower levels of miR-320a-3p in serum were associated with poor overall survival in BC Brazilian patients. In summary, we observed that miR-320a and miR-4433b-5p distinguished BC from controls with high specificity and sensibility, regardless of the sample source. In addition, lower levels of miR-150-5p and higher levels of miR-142-5p were statistically significant biomarkers in tissue, according to TCGA. When combined in panels, all combinations could distinguish BC patients from controls. These results highlight a potential application of these miRNAs as BC biomarkers.

## 1 Introduction

Breast cancer (BC) is the most common malignancy and the second leading cause of death by cancer in women worldwide (Sung et al., 2021). Only in 2020, more than 2 million females had developed the disease, and the occurrence of 66,000 new cases is expected in each year of the triene 2020–2022 in Brazil (INCAdeda.S, 2019). As a heterogeneous disease, different classifications for BC have been proposed, mainly based on histology and risk factors but since the 2000s also based on gene expression. Perou et al. (2000) proposed that the phenotypic variety of BC might be accompanied by a distinct gene expression and described the first molecular classification subdividing tumors expressing hormonal receptors (estrogen and progesterone), overexpressing HER2 oncoprotein, and with the basal phenotype (Perou et al., 2000). This classification was validated and expanded (Sørlie et al., 2001; Farmer et al., 2005; Prat et al., 2013) and adapted to clinical practice by a partly corresponding immunohistochemical (IHC) classification (Goldhirsch et al., 2013). Currently, the molecular classification based on IHC defines four subgroups using four markers, estrogen receptor (ER), progesterone receptor (PR), HER2 expression, and the proliferation marker Ki-67. The subgroups are luminal A (LA), luminal B (LB), HER2 enriched, and triple-negative breast cancer (TNBC). Although the TNBC subgroup is considered a single entity on IHC, it is a very heterogeneous group that reflects on treatment decisions (Marra et al., 2020).

Personalized medicine has been the ultimate goal of current oncology management. Accuracy in the tumor characterization and prediction of patient prognosis based on tumor biology improves the opportunity for target treatments. A better characterization of the genomic landscape, the application of omics technologies, and novel clinical trials will pave the way toward personalized anticancer treatments in breast cancer. Despite the efforts and advances, the morbidity and mortality of BC remain high (INCA, 2019). In this scenario, a deep understanding of BC molecular characteristics is essential to develop new biomarkers for early detection and classification, positively impacting diagnosis, treatment, and effectiveness of controlling this neoplasia.

A class of molecules that have been described to play a significant role in cancer is the microRNAs (miRNAs). miRNAs are small non-coding RNAs that regulate gene expression in biological processes (Ambros, 2004; Bartel, 2009; Ramassone et al., 2018), and their deregulation can lead to cancer development (Rupaimoole and Slack, 2017; Adhami et al., 2018; Mandujano-Tinoco et al., 2018). Several studies suggest that miRNAs can become helpful biomarkers to monitor cancer progression and prognosis (Wang et al., 2016; Adhami et al., 2018; Ozawa et al., 2020a; Hong et al., 2020), but the potential of miRNAs in BC patients remains uncertain. Recently, miR-875 and miR-103a-3p were found as potential prognostic markers in BC patients. Nonetheless, the number of evaluated patients was quite limited, in addition to the absence of a second validation cohort (Liu H. et al., 2022; Liu et al., 2022 X.). Combined circulating miRNAs were validated to accurately distinguish BC patients and subtypes from controls (Kim et al., 2021a; Zhang et al., 2021a; Li et al., 2022b), and to screen BC patients associated with mammography (Zou et al., 2021a; 2022a), highlighting the relevance of the panel’s studies.

Interestingly, a recent study from our group found that lower levels of miR-150-5p, miR-142-5p, and miR-320a in extracellular vesicles from patient serum are associated with advanced tumor grades and larger tumor size (Ozawa et al., 2020b). The authors also identified that a panel comprising miR-142-5p, miR-320a, and miR-4433b-5p could distinguish BC patients from controls with high sensitivity and specificity (Ozawa et al., 2020b). To assess if these miRNAs can also be used as biomarkers in different types of samples, we analyzed the expression of these miRNAs in tumor tissue and cell-free miRNAs (cf-miRNAs) in serum.

## 2 Materials and Methods

This study was approved by the Ethical Committee in Research from the Health Sciences Unit of the Federal University of Paraná (UFPR) (CAAE 19870319.3.0000.0102). All individuals signed a written informed consent form.

### 2.1 Sample Characterization

#### 2.1.1. Fresh Tumor Samples and Serum

We included 30 breast tumor tissues (BC-T) and 29 nontumor adjacent tissues (NT) collected during surgery at the Hospital Nossa Senhora das Graças (Curitiba, Southern Brazil). We also collected peripheral blood (BC-S) from 67 patients before surgery in BD Vacutainer^®^ SST™ II Advance tubes, and we further processed the blood to obtain serum. The tissue samples were stored in RNA Stabilizing Solution (RNAlater^®^—Invitrogen) until processing. In addition, we collected control serum samples (CT) from 19 healthy volunteers at the Federal University of Parana. We excluded controls younger than 50 years or with a previous personal or familial history of cancer and patients with previously neoadjuvant chemotherapy. We obtained clinical and histopathological information about the immunohistochemical markers, age at diagnosis, cancer or death events, histological classification and grade of tumor, the presence or absence of axillary lymph node metastasis, and tumor size from the patient’s medical reports (Table 1). The classification was based on Goldhirsch et al. (2013).

**TABLE 1:** Clinicopathological data obtained from the TCGA database and clinical reports of breast cancer patients.

	TCGA			Brazilian cohort*			
	NT	LA	BLBC	NT	LA	TNBC	CT
N	75	250#	83	29	56	27	19
Median age	57.21 ± 15.67	58 ± 13.4	55 ± 13.06	55 ± 14.86	61 ± 13.13	54 ± 16.11	55 ± 14.86
Survival data, ¥	1913 ± 1,046	1812 ± 1,304	1759 ± 1,061	n.i.	16/26	10/26	n.i.
*Menopausal status,* £							
Pre-	17/53	64/227	14/78	n.i	10/56	10/27	2/19
Post-	35/53	155/227	58/78	n.i	46/56	17/27	17/19
Peri-	1/53	8/227	6/78	n.i			
*Tumor size*							
≤20 mm	--	n.i.	n.i.	15/28	10/22	8/13	--
>20 mm	--	n.i.	n.i.	13/28	12/22	5/13	--
*Histological classification*							
Infiltrating ductal	--	168/250	73/83	--	34/56	25/27	--
Infiltrating lobular	--	54/250	2/83	--	8/56	1/27	--
Mixed ductal and lobular	--	11/250	1/83	--	8/56	0	--
Others †	--	17/250	7/83	--	6/56	1/27	--
*Histological grade*							
I, IA, IB	--	66/248	12/81	--	5/24	0	--
II, IIA, IIB	--	132/248	60/81	--	19/24	3/12	--
III, IIIA, IIIB, IIIC	--	46/248	9/81	--	0	9/12	--
IV, X	--	6/248	0/81	--	0	0	--
*Metastatic axillary lymph node*							
POS	38/69	121/232	26/77	7/28	5/47	10/22	--
NEG	31/69	111/232	51/77	21/28	42/47	12/22	--

N, number of all patients included in the study for each group. (*) The Brazilian cohort includes all the patients who have at least one of the studied samples—serum (BC-S = 67; CT = 19) or tissue (BC-T = 30; NT = 29). NT, adjacent non-tumor tissue; LA, luminal A; TNBC, triple-negative breast cancer; CT, serum samples of controls; BLBC, basal-like breast cancer; (--), not applicable; and (n.i.), not informed. (£) menopausal status of Brazilian patients was estimated based on the age of patients, and patients with peri- and post-menopausal statuses are grouped (≥50). (¥) Survival data for TCGA are represented as days to death, while the Brazilian cohort is the number of patients with data about cancer or death events. (†) includes mucinous carcinoma, tubular carcinoma, medullary carcinoma, and metaplastic carcinoma. Numbers in each parameter differ due to the lack of information for some patients. (#) LA group from the TCGA database includes four male samples, which have been removed from posterior analyses.

#### 2.1.2 TCGA

We evaluated the tissue expression profile in a second cohort using data from 822 samples with miRNA mature strand expression RNA-seq extracted from “The Cancer Genome Atlas” (TCGA) database, from TCGA BRCA cohort version 2017-09-08. TCGA data were obtained as log2 (RPM+1) and converted to fold change (FC). We further processed the data according to adjusted *p*-value < 0.05 and false discovery rate (FDR) < 0.05.

TCGA data contained the following clinicopathological parameters: age of diagnosis, histological classification, grade and size of the tumor, and the presence or absence of axillary lymph node metastasis, in addition to days to death and overall survival information (Table 1). We selected for analysis the intrinsic subtypes luminal A (LA) (*n* = 250) and basal-like breast carcinoma (BLBC) (*n* = 83) on TCGA samples and nontumor samples. We identified the target miRNAs using the unique identification of mature miRNAs (MIMAT ID). The selected miRNAs were as follows: miR-142-5p (MIMAT0000433), miR-150-5p (MIMAT0000451), miR-320a-3p (MIMAT0000510), and miR-4433b-5p (MIMAT0030413) on tumor (BC-T) and nontumor samples (NT). We accessed clinical and histopathological information and performed differential expression analyses comparing NT and BC-T samples in addition to the intrinsic subtypes LA and BLBC.

### 2.2 Sample Processing

We stored all tumor samples in RNA Stabilizing Solution until further processing. We centrifuged the blood samples at 700 g for 10 min to obtain serum. For RNA extraction from tissue, we used the miRNeasy kit (Qiagen, Hilden, Germany), while for RNA from serum, we used the MagMAX™ Total Nucleic Acid Isolation Kit (Thermo Fisher Scientific, Waltham, United States), both according to the manufacturer’s instructions. We then evaluated the quality parameters using the spectrophotometer *NanoDrop 2000* (Thermo Fisher Scientific, Waltham, United States) and stored samples at −80°C until further processed.

### 2.3 RT-qPCR

We performed reverse transcription–quantitative polymerase chain reactions (RT-qPCRs) using a TaqMan MicroRNA Reverse Transcription Kit (Thermo Fisher Scientific, Waltham, United States). Briefly, for a final volume of 20 μl, 10 ng of total RNA extracted was mixed with 1.25 mM dNTPs, 3.75 U/μl of MultiScribe™ Reverse Transcriptase, 1x of Reverse Transcription Buffer, 0.25 U/μl of RNase inhibitor, and 0.125x of each primer—has-miR-142-5p (ID: 002248), has-miR-150-5p (ID: 000473), has-miR-320a (ID: 002277), and has-miR-4433b-5p (ID: 466345_mat). The mixture was submitted to cycles of 25 °C for 10 min, then 37 °C for 2 h, and 85 °C for 5 min on an Eppendorf 5331 MasterCycler Gradient Thermal Cycler (Eppendorf, DE). Next, cDNA samples were diluted at 1:5, and 2.25 μl of this mix was added to 1x TaqMan Universal PCR Master Mix II (no UNG) for a final volume of 5 μl in 384-well plates. Triplicates were performed for each sample, and the median was used for analysis. qPCR was performed using the ViiA 7 Real-Time PCR System (Applied Biosystems, United States) with the following protocol: 50 °C for 5 min, 95 °C for 10 min, and 40 cycles of 95 °C for 15 s, 55 °C for 30 s, and 60 °C for 30 s. The BT-474 ductal carcinoma cell line was used as a calibrator sample among plates. We used the expression of the small-nucleolar RNA RNU48 as the endogenous control. The 2^−ΔΔCq^ method was used to estimate the miRNA expression level using the QuantStudio Real-Time PCR Software v1.3 (Thermo Fisher Scientific, Waltham, United States).

### 2.4 Statistical Analysis

We converted TCGA data obtained as log2 (RPM+1) to fold change (FC). We used the 2^−ΔΔCq^ to calculate the FC values for qPCR analysis. We tested normality using the Shapiro–Wilk normality test and the D'Agostino & Pearson omnibus test in GraphPad Prism 8 (GraphPad Software Inc., United States). We adopted nonparametric tests for data that did not pass either test. We compared groups using the unpaired *t* test, the Mann–Whitney test, or the Kruskal–Wallis test as fitting, followed by Dunn’s multiple comparisons test. We evaluated clinicopathological differences between groups that allow evaluation by presence/absence using Fisher’s exact test (SISA *quantitative skills*). Based on days to death and the presence/absence of death event, we calculated overall survival (OS), comparing low or high expression of each miRNA through log-rank (Mantel–Cox) and the Gehan–Breslow–Wilcoxon tests. We used GraphPad Prism 8 (GraphPad Software Inc., United States) to calculate individual receiver operating characteristic (ROC) curves based on FC values. For combined ROC curves, we performed a binary logistic regression analysis using IBM SPSS Statistics 26.0 (IBM SPSS Statistics Inc., Armonk, NY, United States), and we determined the cutoff, sensitivity, and specificity by Youden’s index (higher sensitivity + specificity).

## 3 Results

### 3.1 miR-320a-3p and miR-4433b-5p Are Overexpressed in Serum Samples and Discriminate Patients From Controls, Especially When Combined in Panels

**FIGURE 1:**
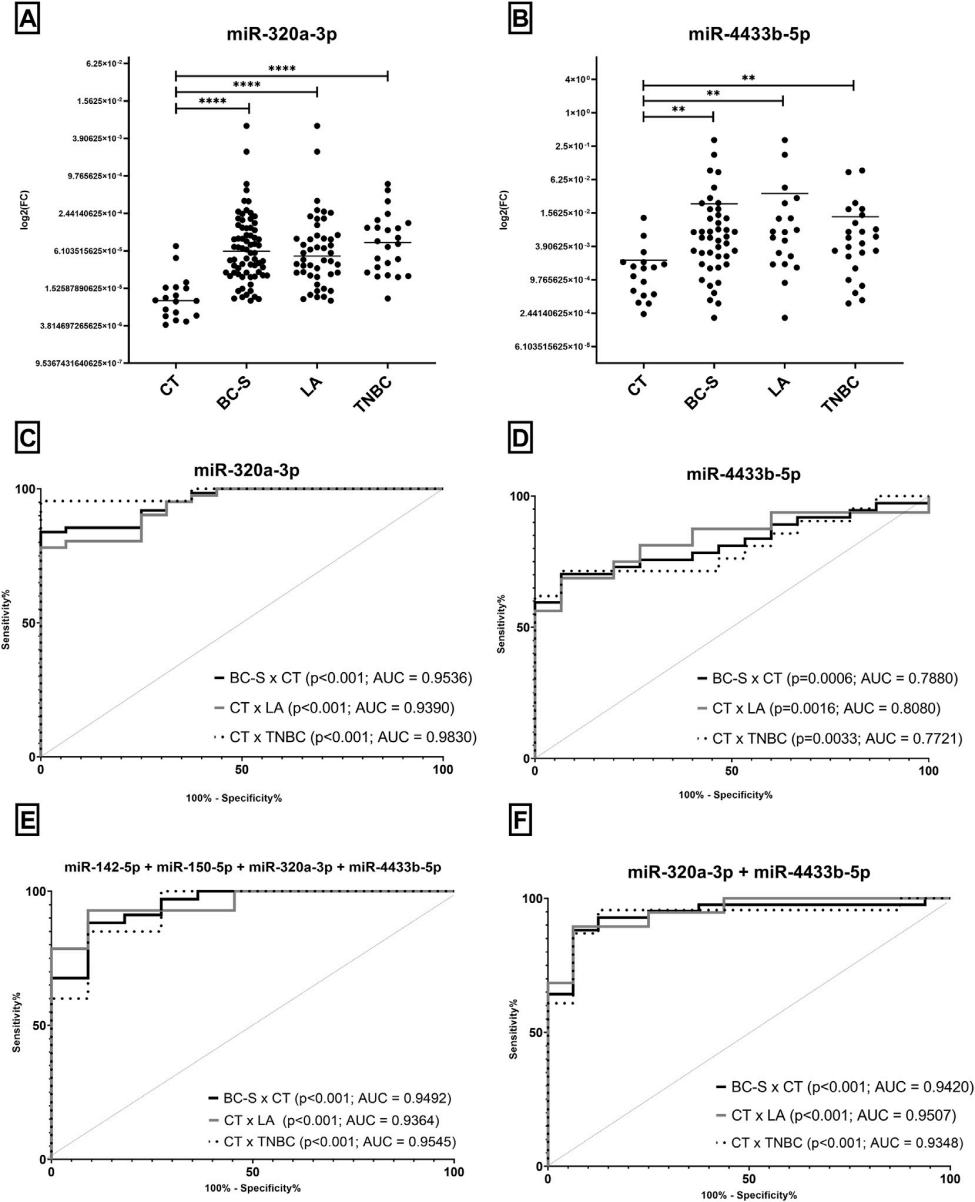
Expression levels of miRNAs by RT-qPCR in serum samples and potential of cf-miRNAs to discriminate BC and subtypes (LA and TNBC) from CT. Expression levels of miRNAs **(A)** miR-320a-3p and **(B)** miR-4433b-5p in BC-S and subtypes (LA and TNBC) and CT. After outlier removal, each dot represents one sample. ROC curves for BC-S diagnosis and prognosis in Brazilian samples **(C–F)**, comparing BC-S to CT (black), CT to LA (gray), and CT to TNBC (dotted). ROC curves were performed to evaluate miR-320a-3p **(C)** or miR-4433b-5p **(D)** individually **(F)** or in combination with all miRNAs in a completed panel **(E)**. ROC = receiver operating characteristic; AUC, area under the curve; CT, serum controls; BC-S, breast cancer serum samples; LA, luminal A; and TNBC, triple-negative breast cancer. (**) *p* = 0.001 and (****) *p* < 0.0001.

We analyzed four miRNAs in 53 serum samples of breast cancer patients (BC-S) and 19 CT. We found higher levels of miR-320a-3p and miR-4433b-5p in BC-S and BC subtypes (LA and TNBC) than in CT (Figures 1A,B). Both miRNAs discriminate BC-S and its subtypes compared to CT with high sensitivity and specificity, either alone or combined in a panel (Figures 1C–F). Interestingly, miR-320a-3p discriminates TNBC to CT with AUC = 0.9830 (Figure 1C). Although miR-150-5p and miR-142-5p revealed no DE in BC-S samples when combined in panels, both miRNAs improved the discrimination of BC-S (including subtypes) from CT samples with high sensitivity and specificity (Figure 1E). No difference was observed in the miRNA expression associated with age, histological grade, size of the tumor, or axillary lymph node status.

### 3.2 In Tissue Samples, Lower Levels of miRNAs Discriminate Tumors From Non-Tumor Samples

**FIGURE 2:**
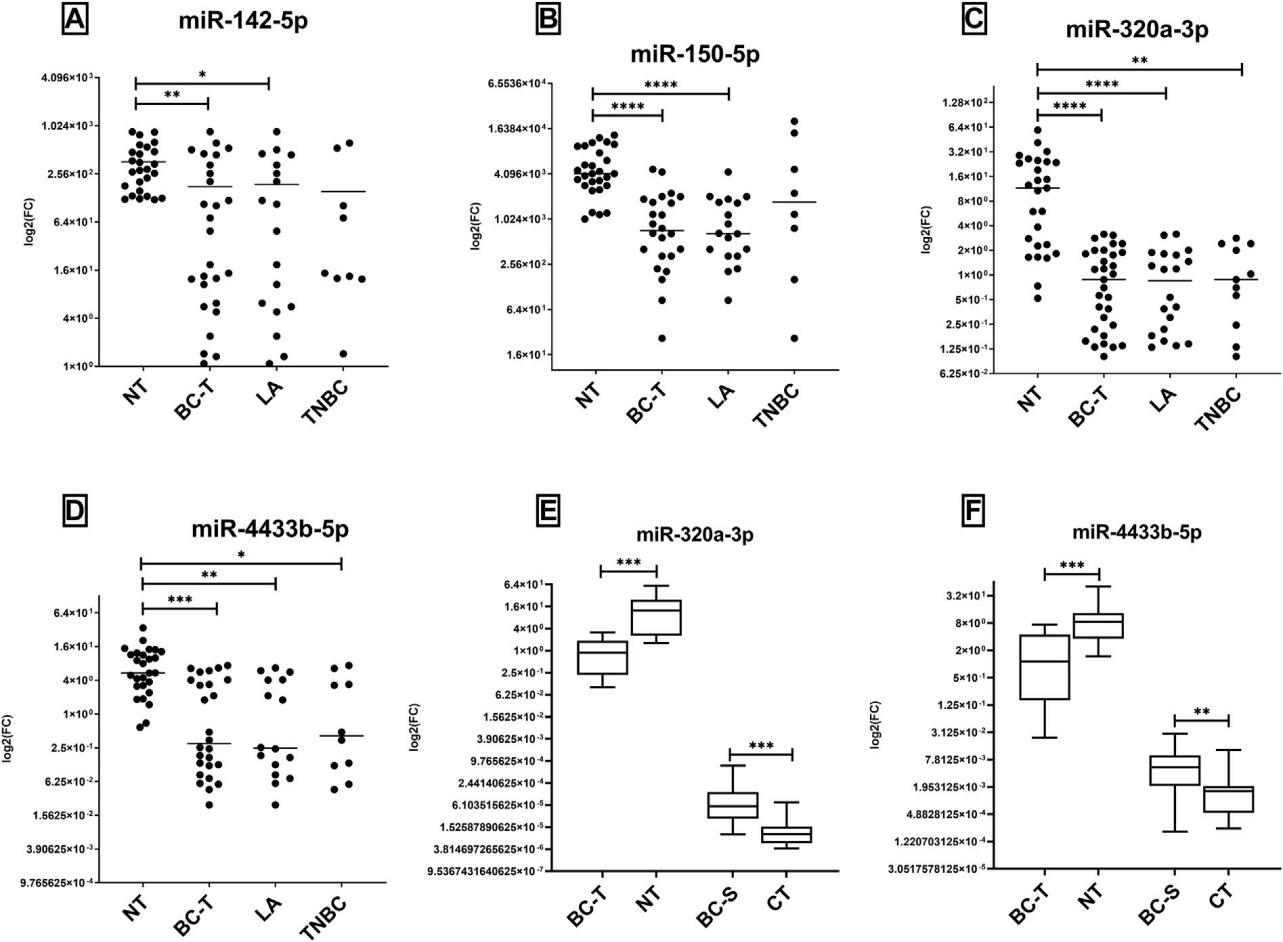
Expression levels of miR-142-5p, miR-150-5p, miR-320a, and miR-4433b-5p on fresh tissue samples. miR-142-5p, miR-150-5p, miR-320a, and miR-4433b-5p evaluated by RT-qPCR in tissue samples **(A–D)**, and the comparison between serum and tissue expression in matched samples **(E,F)**. **(A–D)** Levels of expression were evaluated in NT and BC-T samples; BC-T samples comprised LA and TNBC subtypes, and the expression of all miRNAs was evaluated. After outlier removal, each dot represents one sample in the tissue group. NT samples showed overexpression of all miRNAs evaluated compared to BC-T **(A)** and LA samples. **(E,F)** BC-S and BC-T were evaluated as matched pairs from patients with both samples to all miRNAs (*n* = 16). NT and CT were also compared but not paired. Inverse directions were found between miRNA expression comparing BC-S (high) and BC-T (low). BC-T, breast cancer tissue samples; NT, non-tumor samples; BC-S, breast cancer serum samples; LA, luminal A; TNBC, triple-negative breast cancer; and CT, controls. (*) *p* = 0.01; (**) *p* = 0.001; (***) *p* = 0.0001; and (****) *p* < 0.0001.

In contrast to what we observed in serum, we observed lower expression levels of the four evaluated miRNAs in tissues in BC-T than in NT samples (Figures 2A–D). This trend is also true when we compared tissue sample and serum from the same patient; while miR-320a-3p and miR-4433b-5p were higher in BC than CT in serum, we observed in tissue an opposite trend (Figures 2E,F). We also found higher expression of all miRNAs in NT than LA subtype and overexpression of miR-320a and miR-4433b-5p in NT samples compared to that in TNBC (Figures 2A–D). High or low expression of the miRNAs was not correlated with the clinicopathological parameters evaluated.

We performed ROC curve analysis to investigate the diagnostic potential of miRNAs for BC-T and subtype differentiation. We noticed high sensitivity and specificity by all miRNAs to discriminate BC-T from NT. Of note, a panel combining miR-320a-3p and miR-4433b-5p showed improved values for AUC when comparing BC-T patients to NT, with 100% sensitivity (Table 2).

**TABLE 2:** Data about receiver operating characteristic (ROC) curves to investigate the diagnostic potential of miRNAs on Brazilian tissue samples.

Comparison	miRNA	AUC	Sensitivity	Specificity	*p*-value
*NT x BC*	miR-142-5p	0.7434	66.67	96.15	0.0005
	miR-150-5p	0.8108	91.67	85.71	<0.0001
	miR-320a-3p	0.9009	74.19	81.48	<0.0001
	miR-4433b-5p	0.8462	65.38	81.48	<0.0001
	miR-150-5p + miR-320a-3p panel	0.8929	100.0	67.86	<0.0001
	miR-142-5p + miR-320a-3p panel	0.7232	89.29	46.43	0.0041
	**miR-320a-3p + miR4433b-5p**	**0.9121**	**100.0**	**78.57**	**< 0.0001**
	miR-142-5p + miR-320a-3p + miR-4433b-5p panel	0.9084	100.0	78.57	<0.0001
	miR-150-5p + miR-320a-3p + miR-4433b-5p panel	0.9075	100.0	78.57	<0.0001
	miRNAs complete panel*	0.8982	78.57	100.0	<0.0001
*NT x LA*	miR-142-5p	0.7368	61.11	100.0	0.0063
	miR-150-5p	0.8797	94.44	85.71	<0.0001
	**miR-320a**	**0.9125**	**70.00**	**92.59**	**< 0.0001**
	miR-4433b-5p	0.8482	68.75	81.48	0.0001
	miR-150-5p + miR-320a-3p panel	0.9079	89.29	78.95	<0.0001
	miR-142-5p + miR-320a-3p + miR-4433b-5p panel	0.9082	78.57	100.0	<0.0001
	miR-150-5p + miR-320a-3p + miR-4433b-5p panel	0.9056	78.57	100.0	<0.0001
	miRNAs complete panel*	0.8929	78.57	100.0	<0.0001
*NT x TNBC*	miR-142-5p	0.7571	77.78	100.0	0.0170
	miR-150-5p	0.6473	--	--	0.2092
	miR-320a	0.8799	63.64	92.59	0.0003
	miR-4433b-5p	0.8429	60.00	88.89	0.0015
	miR-150-5p + miR-320a-3p panel	0.9152	92.86	75.00	0.0004
	miR-142-5p + miR-320a-3p + miR-4433b-5p panel	0.9008	78.57	100.0	0.0003
	**miR-150-5p + miR-320a-3p + miR-4433b-5p panel**	**0.9241**	**75.00**	**100.0**	**0.0003**
	miRNAs complete panel*	0.9240	75.00	100.0	0.0003

(*): The four miRNAs were evaluated together. In bold and underlined are the highest AUC values for the group comparison. Only AUC>0.7 is presented, except for NT x TN, using miR-150-5p. Sensitivity and specificity are presented as percentages (%). BC, breast cancer; NT, adjacent non-tumor tissue; LA, luminal A; TNBC, triple-negative breast cancer; and (--) not evaluated.

In addition, all the studied miRNAs distinguished LA or TNBC from NT samples, except for miR-150-5p, which only differentiated the LA group. Finally, it is interesting to note that the highest values of AUC in panels include miR-320a-3p, even though other combinations were just as suitable (Table 2).

### 3.3 Lower Expression Levels of miR-320a-3p in Serum Associated With Poor Overall Survival in the BC Brazilian Cohort

**FIGURE 3:**
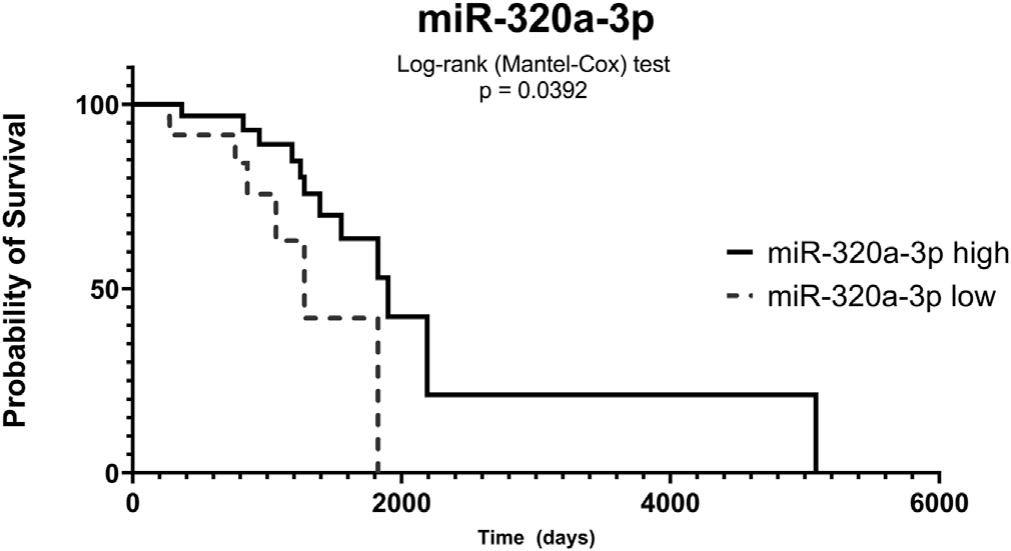
Low expression of miR-320a-3p in serum is correlated with poor overall survival. The serum samples of breast cancer patients (BC-S) were divided into groups based on miR-320a-3p expression levels (high—straight line; low—dotted line). Time was evaluated in days and probability in percentage (%) of survival.

We divided the patients into two groups based on their miRNA median expression to evaluate the influence of these miRNAs on the disease-specific survival of the Brazilian cohort. We compared the high or low expression to the event of death/survival and days to death. We observed that lower expression levels of miR-320a-3p in serum samples were associated with poor overall survival when compared to the group with higher levels (Figure 3).

### 3.4 Differential Expression of miRNAs in BC Samples From TCGA Database

We analyzed data from a total of 822 TCGA samples. Although the median age did not differ among the groups, we observed that 63.6% of BC-T patients had post-menopausal status, compared to 46.7% in the control group. Most BLBC was represented by infiltrating ductal carcinoma (88%), unlike LA, which revealed heterogeneous histology. In addition, we found about a quarter of LA patients with early BC histological grade (stage I), compared to 14.81% on BLBC. In addition, BLBC presented a higher axillary lymph node metastasis frequency than LA (64.4% vs*.* 44.4%, respectively) (Table 1).

**FIGURE 4:**
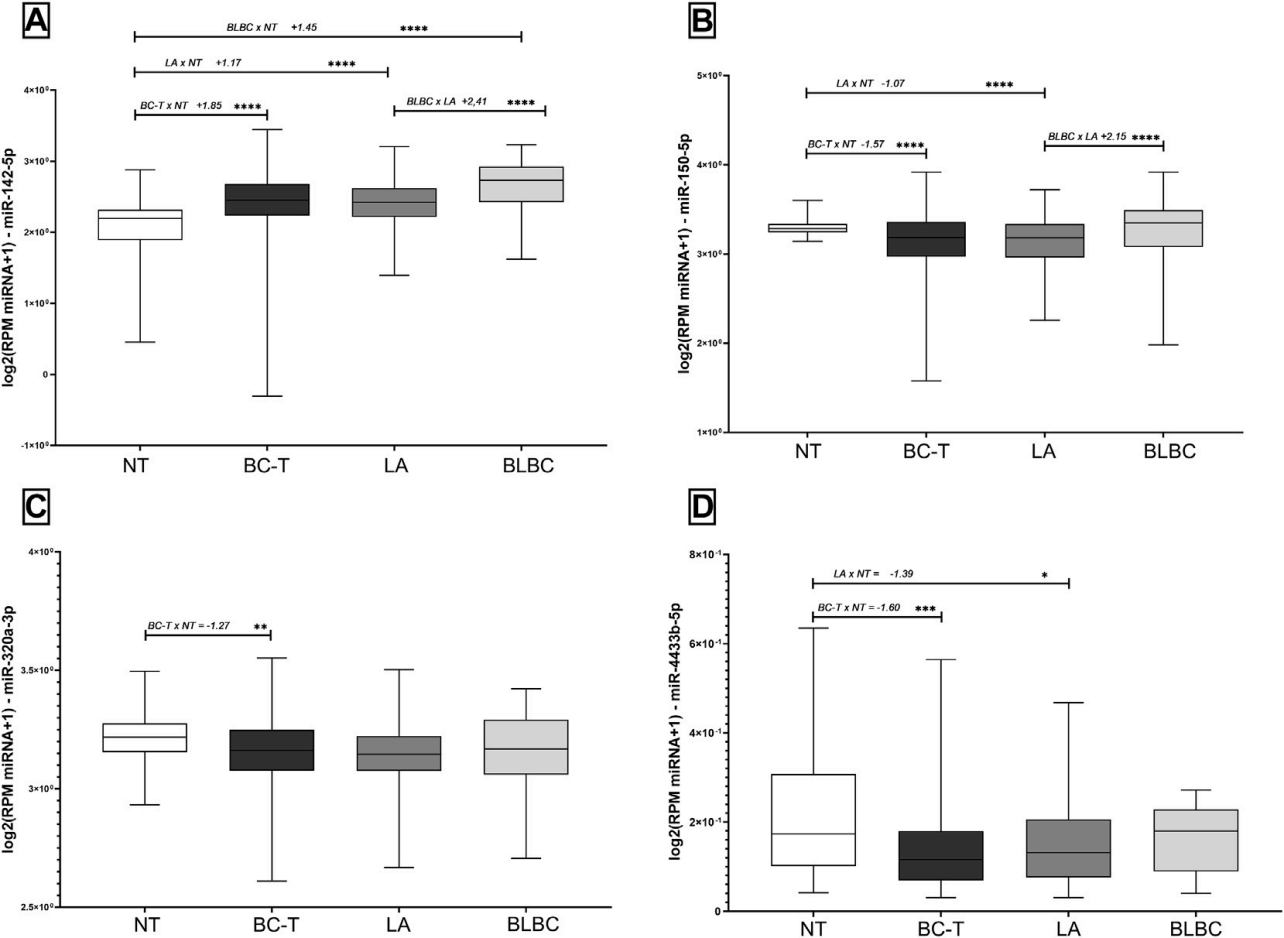
Expression levels of miR-142-5p, miR-150-5p, miR-320a-3p, and miR-4433b-5p in tissue samples according to the TCGA database. Expression levels of **(A)** miR-142-5p, **(B)** miR-150-5p, **(C)** miR-320a-3p, and **(D)** miR-4433b-5p in BC-T and subtypes (LA and BLBC) and NT. Fold change between the two groups compared and the order of comparisons were indicated before the FC and *p* values. NT, non-tumor samples; BC-T, breast cancer tissue samples; LA, luminal A; BLBC, basal-like breast cancer; and FC, fold change. (*) *p* < 0.01; (**) *p* = 0.001; (***) *p* = 0.0001; and (****) *p* < 0.0001.

Similar to the results described for the Brazilian cohort, miR-150-5p, miR-320a-3p, and miR-4433b-5p were downregulated in BC-T samples. On the other hand, tumor and non-tumor comparisons from the TCGA database revealed the overexpression of miR-142-5p in BC-T samples. When analyzing BC subtypes, we observed the overexpression of miR-142-5p and miR-150-5p comparing BLBC *versus* LA. In addition, miR-142-5p showed a higher expression in both BLBC and LA subtypes than in NT samples. On the other hand, we found a reduced expression of miR-150-5p and miR-4433b-5p in the LA subtype compared to that in NT samples. The miRNAs miR-320a-3p and miR-4433b-5p showed no difference between BC subtypes (Figure 4). The expression of all miRNAs was neither correlated with overall survival nor with the clinicopathological parameters evaluated.

### 3.5 The Complete Panel Improved the Diagnostic Value of miRNAs in TCGA Samples

We performed ROC curve analysis to investigate the diagnostic value of miRNAs in TCGA samples. In fact, we found that high levels of miR-142-5p distinguished NT from BC-T, as well as from BC subtypes, with high sensitivity and specificity. Although significant, we observed that the AUC values for miR-150-5p, miR-320a-3p, and miR-4433b-5p were below 0.7 (Table 3). Nonetheless, unlike what we described in the Brazilian cohort (Table 2), in TCGA data, we observed that the complete panel with all four miRNAs studied improved the diagnostic potential of biomarkers in all comparisons performed (Table 3).

**TABLE 3:** Data about receiver operating characteristic (ROC) curves to investigate the diagnostic potential of miRNAs on TCGA samples.

Comparison	miRNA	AUC	Sensitivity	Specificity	*p*-value
*NT x BC*	miR-142-5p	0.7532	65.86	76.00	<0.0001
	miR-150-5p + miR-142-5p panel	0.9317	88.76	86.67	<0.0001
	miR-142-5p + miR-320a-3p panel	0.7681	52.07	89.33	<0.0001
	miRNAs complete panel*	**0.9345**	**87.68**	**90.67**	**< 0.0001**
*NT x LA*	miR-142-5p	0.7371	62.40	76.00	<0.0001
	miR-150-5p + miR-320a-3p panel	0.7458	69.60	70.67	<0.0001
	miR-150-5p + miR-142-5p panel	0.9179	84.80	86.67	<0.0001
	miR-142-5p + miR-320a-3p panel	0.7738	72.00	72.00	<0.0001
	miRNAs complete panel*	**0.9266**	**85.20**	**90.67**	**< 0.0001**
*NT x BLBC*	miR-142-5p	0.8671	72.29	93.33	<0.0001
	miR-150-5p + miR-142-5p panel	0.9680	93.90	92.00	<0.0001
	miR-142-5p + miR-320a-3p panel	0.8694	70.73	92.00	<0.0001
	miRNAs complete panel*	**0.9689**	**93.90**	**92.00**	**< 0.0001**
*LA x BLBC*	miR-142-5p	0.7220	72.29	67.60	<0.0001
	miR-150-5p + miR-142-5p panel	0.7711	68.67	78.80	<0.0001
	miR-142-5p + miR-320a-3p panel	0.7274	59.04	80.80	<0.0001
	miRNAs complete panel*	**0.7728**	**63.86**	**80.80**	**< 0.0001**

Only AUC>0.7 is presented. In bold and underlined are the highest AUC values for the group comparison. (*): the four miRNAs were evaluated together. Sensitivity and specificity are presented as percentage (%). BC, breast cancer; NT, non-tumor tissue; LA, luminal A; and BLBC, basal-like breast cancer.

### 3.6 miRNA Expression Levels Showed an Opposite Direction in Serum (Cell-Free and EVs) Compared to Tissue Samples

We compared the expression levels of all miRNAs in serum and tissue samples (both by TCGA and by RT-qPCR) with our previous results in EVs (Ozawa et al., 2020c). We found the same expression pattern in serum samples compared to our earlier findings in EVs and an opposite expression pattern in TCGA data and fresh tissue samples for most comparisons. There were a few exceptions, mainly for miR-142-5p (Table 4). In addition, we found miR-320a and miR-4433b-5p with a higher expression in TNBC than in CT samples, contrasting with our previous results.

**TABLE 4:** Comparison of the expression level and performance of miR-142-5p, miR-150-5p, miR-320a, and miR-4433b-5p in tissue and serum samples (cell-free miRNAs and EVs).

	TCGA	Fresh tissue	Serum samples	EV-miRNAs#
*BC x CT*				
miR-142-5p	high, AUC = 0.7532	low, AUC = 0.7434	n.s.	high, AUC = 0.7964
miR-150-5p	low, AUC = 0.6552	low, AUC = 0.8108	n.s.	n.s.
miR-320a	low, AUC = 0.6219	low, AUC = 0.9009	high, AUC = 0.9536	high, AUC = 0.8063
miR-4433b-5p	low, AUC = 0.6772	low, AUC = 0.8462	high, AUC = 0.8198	high, AUC = 0.7964
miR-142-5p + miR-320a	low, AUC = 0.7681	low, AUC = 0.7232	high, AUC = 0.9468	high, AUC = 0.9410
miR-142-5p + miR-320a + miR-4433b-5p	low, AUC = 0.6796	low, AUC = 0.9075	high, AUC = 0.9429	high, AUC = 0.8387
*LA x CT*				
miR-142-5p	high, AUC = 0.7371	low, AUC = 0.7368	n.s.	high, AUC = 0.9180
miR-150-5p	low, AUC = 0.6852	low, AUC = 0.8797	n.s.	n.s.
miR-320a	low, AUC = 0.6695	low, AUC = 0.9125	high, AUC = 0.9390	high, AUC = 0.8828
miR-4433b-5p	low, AUC = 0.6279	low, AUC = 0.8482	high, AUC = 0.8375	high, AUC = 0.8672
miR-142-5p + miR-320a-3p	high, AUC = 0.7738	low, AUC = 0.9082	high, AUC = 0.9667	high, AUC = 0.9410
*TNBC/BLBC x CT*				
miR-142-5p	high, AUC = 0.8671	n.s.	n.s.	n.s.
miR-150-5p	n.s.	n.s.	n.s.	n.s.
miR-320a	n.s.	low, AUC = 0.8799	high, AUC = 0.9830	n.s.
miR-4433b-5p	n.s.	low, AUC = 0.8429	high, AUC = 0.8063	n.s.
*LA x TNBC/BLBC*				
miR-142-5p	low, AUC = 0.7220	n.s.	n.s.	high, AUC = 0.9208
miR-150-5p	low, AUC = 0.6322	n.s.	n.s.	high, AUC = 0.8667
miR-320a	n.s.	n.s.	n.s.	n.s.
miR-4433b-5p	n.s.	n.s.	n.s.	n.s.

#EV-miRNAs were evaluated by Ozawa (2020). Only the data that were comparable between the two studies are presented. AUC, area under the curve; n. s, not significant; BC, breast cancer; CT, control samples; LA, luminal A; TNBC, triple-negative breast cancer; and BLBC, basal-like breast cancer.

For miR-142-5p, we found no differential expression in serum. According to the TCGA database, we found higher levels in BC-T than in NT, but in fresh tissue from Brazilian samples, we found an opposite expression pattern. IN addition to that, following Ozawa’s findings, miR-142-5p could discriminate BC-T from NT samples (AUC >0.7) with sensitivity and specificity. In addition, by combining panels, the diagnostic potential was improved.

## 4 Discussion

The value of miRNAs as cancer biomarkers has been studied and discussed for some time, and an increasing number of cancer-associated miRNAs have been identified, including in BC (Bao et al., 2019; Li et al., 2020; Jang et al., 2021). The potential diagnostics of circulating miRNAs, especially from exosomes (EV-miRNAs), has already been discussed (Liu et al., 2019; Ozawa et al., 2020b, 2020a). In addition, other non-coding RNAs (nc-RNAs) are emerging as potential biomarkers as long non-coding RNAs (Gradia et al., 2017; Barazetti et al., 2021; Mathias et al., 2021) and circular RNAs (circ-RNAs) (Qian et al., 2018; Ameli-Mojarad et al., 2021; de Palma et al., 2022). Ameli-Mojarad et al. (2021) showed a higher expression of circRNAs in BC tissues than in adjacent tissues. So, we have a world of new molecules to explore, and the combination of them in different panels must be considered.

Combining circulating miRNAs in panels shows improvement in the diagnosis and prognosis potential. Recently, Turkistani et al. (2021) found that panels of deregulated miRNAs showed a discriminatory potential based on TNBC tumor size, lymph node metastasis, and recurrence status of the disease. Recently, miR-875 and miR-103a-3p were described as potential prognostic markers in BC patients. Nonetheless, the number of evaluated patients was quite limited, in addition to the absence of a second validation cohort (Liu H. et al., 2022; Liu et al., 2022 X.). Combined circulating miRNAs were validated to accurately distinguish BC patients and subtypes from controls (Kim et al., 2021b; Zhang et al., 2021b; Li et al., 2022b) and to screen BC patients associated with mammography (Zou et al., 2021b; 2022b), highlighting the relevance of panel studies. A recent study found that a panel comprising four EV-miRNAs (miR-9, miR-16, miR-21, and miR-429) presented high sensitivity to discriminate BC subtypes of the early stages of the disease. Interestingly, these miRNAs were chosen using the TCGA database (Kim et al., 2021b), drawing attention to the relevance of candidate validation, especially when combined in panels.

A previous study from our group showed the potential of an EV-miRNA panel including miR-142-5p, miR-150-5p, and miR-320a discriminating BC patients from controls with 93.33% sensitivity and 68.75% specificity. In addition, miR-142-5p levels were associated with clinicopathological parameters, such as bigger tumor size, higher stage, and presence of lymph node metastasis (Ozawa et al., 2020b). Aiming to investigate if these miRNAs also have a good performance as biomarkers in different types of samples, we performed a dual sample analysis strategy: TCGA database in tissue and RT-qPCR of miR-142-5p, miR-150-5p, miR-320a, and miR-4433b-5p in tissue and serum samples.

In this work, we found higher levels of miR-320a and miR-4433b-5p in BCS and in the LA subtype than in CT, similar to Ozawa’s results (Ozawa et al., 2020b). In addition, the panel including miR-142-5p, miR-320a, and miR-4433b-5p discriminated BC patients from controls with likewise high sensitivity and specificity. In contrast, lower expression levels of miR-150-5p, miR-320a-3p, and miR-4433b-5p were observed in BC-T than in NT samples, both by TCGA and RT-qPCR analyses of our Brazilian cohort. These miRNAs showed potential diagnostic value in the Brazilian cohort to discriminate BCT from NT samples with higher sensitivity and specificity, either alone or combined in panels. This potential was also observed in TCGA samples, especially in the panel including all four miRNAs (Table 4).

Discussing our results, the dysregulation of miR-320a has been previously described in breast cancer, with an increased expression, suggesting it as a biomarker for invasive disease (Yang et al., 2014). However, its anti-oncogenic potential has also been studied before (Lü et al., 2015; Wang et al., 2015; Yu et al., 2016). Interestingly, in this study, we found significantly low expression levels of miR-320a in BC, both by TCGA and RT-qPCR, strengthening the potential of this miRNA as a biomarker for BC. In fact, miR-320a-3p showed that it could significantly discriminate BC-T from NT tissue (AUC = 0.9009), and this AUC value can be improved when combining miR-320a-3p in panels with other miRNAs. In addition, miR-320a-3p differentiates LA or TNBC subtypes from NT in the Brazilian cohort. In serum, we observed that high levels of miR-320a-3p in BC-S compared to controls can effectively distinguish these groups with higher sensitivity and specificity, according to our previous findings in EV-miRNAs (Ozawa et al., 2020c). Indeed, we found lower levels of miR-320a-3p on BC-S associated with poor overall survival in the Brazilian cohort, highlighting its potential as a diagnostic biomarker.

The literature regarding miR-4433b-5p is quite limited but indicates a trend for its association with cancer. Wu et al. (2019) observed a reduction in BCR-ABL mRNA through miR-4433 regulation. Ozawa et al. (2020b) found that miR-4433-5p, which was also part of the miRNA panel to distinguish LA from CT samples, was overexpressed in BC patients compared to that in CT. We noticed reduced levels of this miRNA in BCT compared to those in NT samples both by RT-qPCR and TCGA, and it showed high sensitivity and specificity as a BC biomarker. In addition, a combined panel including miR-4433b-5p and miR-320a-3p improved their diagnostic potential. Interestingly, we observed increased expression levels of miR-4433b-5p in serum similar to what was found in EVs (Ozawa et al., 2020c). These results indicate a potential involvement of miR-4433b-5p in mediating cell-to-cell communication in BC.

We found no differential expression of miR-142-5p and miR-150-5p in serum, contrasting with our previous EV results (Ozawa et al., 2020b) (Table 4). Tissue samples showed reduced expression of both miRNAs in BC-T and LA subtype compared to that in NT samples. Our RT-qPCR experiments showed a lower expression level of miR-142-5p in BCT samples, but the TCGA database showed overexpression of this miRNA. Likewise, the cancer literature about miR-142-5p is controversial, including in BC. Overexpression of miR-142-5p was previously found in BC tissue and was also associated with increased tumor size and metastasis, suggesting that miR-142-5p could be a possible target therapy for BC (Xu and Wang, 2018; Yu et al., 2019). On the other hand, a recent study found miR-142-5p acting as a tumor suppressor in BC, inhibiting cell invasion and migration by targeting DNMT1 (Li et al., 2022a). Lower levels of miR-142-5p in BC were also found to be negatively correlated with circWAC, another type of non-coding RNA (Wang et al., 2021).

Some authors found a reduced expression of miR-142 in BC samples but in a mature miRNA generated from the 3p arm of the precursor (miR-142-3p) (Mansoori et al., 2019; Ma et al., 2020; Xu et al., 2020). Nonetheless, we found miR-142-5p as a potential diagnostic biomarker in the Brazilian cohort, with a reduced expression in BC compared to that in NT samples. We also found lower levels of miR-142-5p in the LA subtype than in the BLBC according to TCGA samples and higher levels of this miRNA in the LA subtype than in the TNBC according to studied EV-miRNA samples (Table 4). When miR-142-5p was combined in panels in the Brazilian cohort, the diagnostic potential and sensitivity improved, similar to what was described for EVs (Ozawa et al., 2020c).

TNBC is a heterogeneous group of tumors and comprises at least six different subtypes, including basal-like breast carcinoma (BLBC) (Millikan et al., 2008; Garmpis et al., 2020; Marra et al., 2020). In our study, similar to what was described for TNBC (Bou Zerdan et al., 2022; Derakhshan and Reis-Filho, 2022), BLBC comprised mainly of infiltrating ductal carcinoma, presenting higher metastatic axillary lymph nodes than the LA subtype.

miR-150 seems to be involved in the tumorigenesis and development of a few solid tumors, but the role of this miRNA remains controversial (Wang et al., 2016; Kim et al., 2017; Koshizuka et al., 2018; Xiao et al., 2019). Some studies found that overexpression of miR-150-5p could inhibit apoptosis and increase EMT and cancer progression (Huang et al., 2013; Lu et al., 2019). However, miR-150-5p′ targets were previously associated with cancer growth and metastatic events (Jiang et al., 2019; Wang et al., 2019; Jia et al., 2021), while miR-150-5p overexpression has been described to be associated with reduced tumor aggressiveness. Similarly, the overexpression of miR-150-5p in BC cells has already been associated with decreased proliferation, invasion, and migration properties (Hu et al., 2019; Jiang et al., 2019; Jia et al., 2021). In our study, TCGA analysis showed that BLBC had an overexpression of miR-150-5p compared to that of LA. In the Brazilian cohort, we found reduced expression levels of miR-150-5p in the LA subtype compared to those in NT samples (AUC = 0.8797). In addition, the diagnostic potential of miR-150-5p improved when combined in panels, especially with miR-320-3p (AUC = 0.9079), suggesting these miRNAs as potential biomarkers to identify the LA subtype.

In summary, the present study showed high expression of miR-320a-3p and miR-4433b-5p in serum from BC patients, in accordance with our previous results on EVs. In contrast, we found reduced levels of miR-142-5p, miR-150-5p, miR-320a-3p, and miR-4433b-5p in tumor tissues from BC patients. Nevertheless, all miRNAs discriminated BC and LA subtypes from NT tissue with high sensitivity and sensibility. In serum samples, we observed that miR-320a-3p and miR-4433b-5p could distinguish BC and LA from CT. In addition, the different combinations of miRNAs in panels improved the diagnostic potential of BC patients and subtypes compared to that of controls. Finally, we found lower levels of miR-320a-3p associated with poor overall survival. Overall, we suggest that the studied miRNAs have potential as diagnostic biomarkers for BC when compared to that for controls and discriminate the LA subtypes. The small number of patients in this study is a limitation, and additional studies in larger samples and also testing new combinations of miRNAs and other classes of ncRNAs will be needed to address the role of these miRNAs in BC tumorigenesis and progression and their use to access the diagnostic, classification, and prognosis.

## Data Availability

The datasets presented in this study can be found in online repositories. The names of repository/repositories and accession number(s) can be found in the main article.
